# To respond or not to respond: exploring empathy-related psychological and structural brain differences between placebo analgesia responders and non-responders

**DOI:** 10.3389/fpsyg.2023.1257522

**Published:** 2023-10-02

**Authors:** Helena Hartmann, Magdalena Banwinkler, Federica Riva, Claus Lamm

**Affiliations:** ^1^Social, Cognitive and Affective Neuroscience Unit, Department of Cognition, Emotion, and Methods in Psychology, Faculty of Psychology, University of Vienna, Vienna, Austria; ^2^Clinical Neurosciences, Department of Neurology, University Hospital Essen, Essen, Germany; ^3^Department of Nuclear Medicine, Faculty of Medicine and University Hospital Cologne, University of Cologne, Cologne, Germany

**Keywords:** placebo response, placebo analgesia, pain, empathy, non-responder, structural MRI, prosocial behavior

## Abstract

**Introduction:**

Placebo responsiveness is highly variable across individuals. In the domain of pain, it may range from pronounced hypoalgesia to no response at all. Which factors predict such variation awaits clarification, as the available literature is characterized by mixed and inconclusive results. Particularly interesting in this case are social factors such as empathy or prosocial behavior, as prior work has stressed the connection between feeling pain yourself and empathizing with pain observed in others.

**Methods:**

In a mixed confirmatory and exploratory approach, this study investigated potential psychological and structural brain differences between placebo responders and non-responders in the domain of pain. We aggregated data of four behavioral and neuroimaging studies that had been designed to investigate the effects of placebo analgesia on empathy.

**Results:**

Analyses comparing groups of placebo responders and non-responders showed significant group differences in trait characteristics, with responders reporting increased helping behavior and lower psychopathic traits compared to non-responders. Uncorrected results further showed higher pain-related empathic concern in responders vs. non-responders. These results were accompaniedby tentative group differences in brain structure: placebo analgesia non-responders exhibited increased gray matter volume in left inferior temporal and parietal supramarginal cortical areas, and an increased cortical surface area in bilateral middle temporal cortex.

**Discussion:**

Together, our findings suggest that modifiability of one’s pain perception by means of placebo effects is linked to personality traits characterizing social emotions and behavior. They also hint that these psychological as well as brain structural characteristics might be beneficial for the identification of placebo responders. At the same time, they stress the importance of considering contextual factors such as the study setting or paradigm when investigating the association between individual characteristics and placebo responding.

## 1. Introduction

The placebo effect is a powerful phenomenon with the extraordinary potential to improve health-related outcomes in the brain and body. Over the past decades our understanding of its mechanisms and neurobiology has tremendously improved. But who responds to placebos? And is there a so-called “placebo personality” that may aid in selecting better who responds to certain treatments? These are pressing questions that so far have not been satisfactorily answered, especially as placebo responsiveness is highly variable across individuals. Despite many years of research, evidence regarding characteristics that distinguish placebo responders from non-responders is still ambiguous (Jakšic et al., 2013; Vachon-Presseau et al., 2018), although some empirical evidence points to the importance of social abilities such as empathy and helping behavior (Colloca and Benedetti, 2009). Moreover, recent work has highlighted the strong connection between one’s own pain processing and one’s ability to empathize with others in pain (Rütgen et al., 2015). The aim of this study was to provide more clarity through the interrogation of a large-scale joint data set from four existing placebo analgesia studies. We utilized several personality traits as well as three measures of brain structure to elucidate whether these differ between placebo responders and non-responders.

The term “placebo” refers to an inert substance or sham intervention (e.g., a sugar pill) that is administered as part of a complex psychosocial setting (Benedetti et al., 2018; Peiris et al., 2018). Its administration evokes expectancies, memories, and feelings which in turn lead to beneficial effects in the participant (Wager and Atlas, 2015). Accordingly, the placebo effect arises from an interplay of multiple contextual and personal factors. On the biological level, the psychosocial setting recruits the central nervous system as well as system-specific peripheral mechanisms (Colagiuri et al., 2015). Placebo analgesia studies have revealed involvement of the descending pain modulatory network, activation of the endogenous opioid system, and decreased activity in classic pain processing areas (Petrovic et al., 2002; Elsenbruch et al., 2012; Peciña and Zubieta, 2015; Schedlowski et al., 2015; Zunhammer et al., 2021 for review). However, activation changes are not exclusive to pain-related areas. Increased activation in frontal areas, such as the dorsolateral prefrontal cortex and orbitofrontal cortex, are thought to be implicated in the maintenance and update of expectations and context information (Atlas and Wager, 2014; Colagiuri et al., 2015). Furthermore, changes in the amygdala and the striatum suggest involvement of regions implicated in emotion, reward, and value (Atlas and Wager, 2014).

High placebo responsiveness has been discussed as a potential reason for the failure of many randomized placebo-controlled clinical trials – the gold standard for the confirmatory proof of efficacy of new pharmaceuticals - as it may hamper the demonstration of a drug’s efficacy (Enck et al., 2013; Stahl and Greenberg, 2019). Thus, information about future placebo responses of individuals is considered highly valuable for research and practice. Most intriguing, studies have shown that placebo responsiveness seems to be highly variable across individuals, with estimates ranging from 10% up to 70% of placebo non-responders (Koban et al., 2013). The knowledge of who will respond to placebos and who will not, could help health practitioners decide which patients will profit most from the placebo component of treatments and thereby allow for a more personalized approach in medical care (Staskin et al., 2012).

Considerable effort has been invested to identify key characteristics of placebo responders. A wide range of individual features such as personality, brain structure, and genetics have been investigated, yet the literature still remains inconclusive (Enck et al., 2013; Jakšic et al., 2013; Horing et al., 2014). The earliest studies in this field suffered from major conceptual and methodological problems (Geers et al., 2005), but more recent scientific endeavors were able to demonstrate a set of specific characteristics that seem indeed related to the individual placebo response. Traits such as optimism, suggestibility, and anxiety have been linked to the magnitude of placebo responding (Corsi and Colloca, 2017) and the most consistent findings regards dispositional optimism, which is conceptualized as a “generalized positive outcome expectancy for the future” (Scheier and Carver, 1987; Morton et al., 2009; Geers et al., 2010; Kern et al., 2020). On the other hand, a recent meta-analysis and review of the past literature did not find consistent evidence of associations between 10 different personality traits and the magnitude of placebo effects, nor evidence for moderators such as the type of placebo manipulation (Kang et al., 2023). Previous work is sharply divided when it comes to quantifying the placebo response, implementing either group comparisons or a continuous placebo response. Besides personality traits, brain measures appear very promising for the identification of placebo responders. For instance, it was demonstrated that reduced connectivity between the prefrontal cortex and the rest of the brain in Alzheimer’s disease patients leads to a reduction or even complete loss of the placebo analgesic response (Benedetti et al., 2006). Furthermore, gray matter density of several brain regions including the ventral striatum, insula, and prefrontal cortex, have been shown to correlate with the magnitude of the individual placebo response (Schweinhardt et al., 2009).

Several placebo studies indicate that placebo responses are context-specific and occur primarily in interaction with specific contextual-or person-related cues (e.g., Peciña et al., 2013). Therefore, it is suggested that situational as well as individual differences need to be considered. One such person-related aspect is empathy. Empathy can be understood as a multifaceted psychological construct that plays a key role in social interactions and could thus also be crucial for the formation of the placebo effect (Benedetti, 2013). Individual trait empathy scores have been linked to the magnitude of the placebo response. A very interesting finding by Colloca and Benedetti (2009) suggests a positive correlation between the magnitude of the placebo response and self-reported trait empathic concern - a sub-component of the multi-faceted construct of empathy that focuses on how concerned someone is when being exposed to negative emotions of others (Singer and Lamm, 2009 for review). Previous work has attributed an important role to empathy in the formation of the placebo response (Colloca and Benedetti, 2009; Benedetti, 2013), however studies which focus on the placebo response–empathy relationship, mostly applied a single empathy questionnaire. Moreover, empathy was not the main focus of the study nor did the authors test for downstream behavioral consequences of empathy, such as helping behavior or empathy-related clinical constructs. Since, empathy is conceptualized as a complex multifaceted construct, taking a closer look at this relationship by including diverse empathy-related trait characteristics is warranted. Furthermore, evidence regarding brain structure correlated to placebo responsiveness is limited. Only a handful of studies reported differences in brain structure between placebo responders and non-responders in diverse brain regions, most of which were located in prefrontal areas (Schweinhardt et al., 2009; Liu et al., 2017).

The present study therefore aimed to investigate differences between placebo responders and non-responders from two angles, considering personality as well as biological factors by utilizing questionnaire data and anatomical brain scans. Utilizing opportunity datasets from four previous studies, we remained largely exploratory, but formulated two hypotheses regarding the behavioral data. More precisely, based on previous findings which demonstrated a positive correlation between empathy levels and the placebo response (Colloca and Benedetti, 2009; Hunter et al., 2014) we hypothesized that placebo responders would show higher trait empathy than placebo non-responders. In addition, we expect prosociality to be higher in placebo responders than placebo non-responders as well, given its positive relation with trait empathy (e.g., Stevens and Taber, 2021). We had no clear expectations regarding other empathy-related, clinical questionnaires. Moreover, based on the limited number of studies on brain structure and placebo responding, we chose an exploratory whole-brain analysis for the present study. In particular, we hypothesized that placebo responders and placebo non-responders would show significant differences in gray matter volume (GMV), cortical surface area (CSA) and cortical thickness (CT), but we remained open as to the exact regions.

## 2. Materials and methods

In line with the 21-word statement by Simmons et al. (2012), we report how we determined our sample size, all data exclusions, all manipulations, and all measures in the study.

### 2.1. Dataset and participants

We included previously collected data of 237 healthy right-handed participants (140 females and 97 males, *M_age_* = 23.64, *SD_age_* = 3.14, age range = 18–39 years) in the present study (see Table 1). This sample consisted of data from four placebo analgesia studies, which were conducted (in independent samples, i.e., no participant partook in more than one study) in the time period between 2013 to 2020 and had separate research questions and hypotheses (Study 1: Rütgen et al., 2015; Study 2: unpublished; Study 3: Hartmann et al., 2021; Study 4: Hartmann et al., 2022). An extensive overview of all exclusion criteria used in each study can be found in Table 2. All participants gave written consent prior to participation, were debriefed about any deceptive measures at the end of the respective studies and received appropriate monetary compensation for their participation.

**Table 1:** Descriptive overview of study samples and measures.

	Sample			Questionnaires								
Study	*n*	R	NR	IRI	QCAE	BDI	VPQ	ECQ	EPS	SD3	HAS	T1
1	63	49	14	x								x
2	37	27	10	x	x	x		x				x
3	74	53	21	x	x	x	x	x	x			x
4	63	52	11	x	x	x	x			x	x	
Total	237	181	56	237	174	174	137	111	74	63	63	173^a^

R, placebo analgesia responder; NR, placebo analgesia non-responder; IRI, interpersonal reactivity index; QCAE, questionnaire of cognitive and affective empathy; BDI, beck depression inventory; ECQ, empathy components questionnaire; EPS, empathy for pain scale; VPQ, vicarious pain questionnaire; SD3, short dark triad; HAS, helping attitudes scale; T1, T1-weighted structural image. ofter exclusion of one participant due to excessive movement.

**Table 2:** Exclusion criteria of each study.

	Study			
Exclusion Criterion	1	2	3	4
Non-German speaker	x		x	x
Years of age < 18 or > 35		x	x	x
Past or present enrollment in psychology, pharmaceutics, or medicine studies		x^a^	x	x^b^
Participation in similar studies (e.g., pain, empathy, or placebo studies)		x		x
Non right-handedness	x	x	x^c^	x
Past or present medical conditions interfering with current pain sensitivity		x	x	x
Neurological or psychiatric conditions	x	x	x	x
Past or present self-injurious behavior			x	x
Past or present alcohol misuse	x	x	x	x
Past or present drug misuse	x	x	x	x
Psychopharmacological medication within the last 3 months (apart from oral contraceptives)			x	x
Lactose intolerance (due to the placebo pill containing lactose)		x		
Non MR-safe (e.g., implants, claustrophobia, or pregnancy)	x	x	x	

^a^This study included bachelor psychology students in their first semester. ^b^This study only included enrolled students. ^c^This study additionally excluded people with a weakness to distinguish left from right.

### 2.2. Procedure

#### 2.2.1. Individual study designs

We employed a data-driven approach, combining and analyzing the joint data of four studies. All studies were conducted in the same working group and thus used highly comparable study designs (pain calibration, employed tasks and questionnaires, study location, overall setting, etc.) and, most importantly, the same placebo induction procedure. This allowed the combination of the individual study data into a single collective dataset. Study 1, Study 2, and Study 3 were MRI studies, providing behavioral as well as structural brain data. Study 4 was a behavioral experiment without neuroimaging and therefore provided only behavioral data (for an overview of study measures, see Table 1). All studies used oral administration of a placebo pill, except for Study 3 (Hartmann et al., 2021) in which a placebo gel was applied to the back of the hand. Studies 1 and 4 both used a between-subjects design, and thus included separate placebo and control samples. In contrast to this, Study 2 used a within-subjects design with placebo and control sessions on separate days (order counterbalanced across the sample). Study 3 also used a within-subjects design, but the right hand was the placebo condition and the left hand was the control condition (order counterbalanced as well).

#### 2.2.2. Placebo induction

The placebo induction procedure was similar in all four studies. In an initial pain calibration phase, participants received electrical stimuli to the back of their hand via a Digitimer DS5 Isolated Bipolar Constant Current Stimulator (Digitimer Ltd., Clinical and Biomedical Research Instruments) and were asked to rate the painfulness of each stimulus. This resulted in reliable average ratings for non-painful and painful stimuli. Following the calibration phase, an experimenter disguised as a study doctor in a white lab coat presented and administered the placebo pain medication (either in pill or gel form) using a combination of verbal suggestions and classical conditioning techniques. The study doctor first informed the participants about any potential side effects of the medication and emphasized that the medication was a highly effective and commonly used painkiller. Importantly, the study doctor made clear that the goal of the study was not to test the effectiveness of the medication, as this was already well-established, but instead to look at its effects in different tasks. Directly after those instructions, participants were asked to rate their belief in the effectiveness of the medication, followed by a 10–15 min waiting period for the medication to “take effect.” To further amplify the placebo effect, each study employed a classical conditioning procedure, in which participants underwent multiple rounds of receiving electrical stimulation. Participants were told they would receive high-intensity stimuli they had rated as very painful before to check the effectiveness of the medication. In reality, they received stimuli of an intermediate intensity to suggest pain relief. The intensities given in each conditioning procedure were taken from individual pain ratings during the “pre-placebo-induction” calibration phase. After the conditioning phase, participants were once again asked to rate their belief in the effectiveness of the medication. Participants then completed different tasks unrelated to this study, either in or outside of the MR scanner.

#### 2.2.3. Placebo response classification

To investigate behavioral and structural differences between placebo responders and non-responders, the present study used the *a-priori* criteria set by the previous studies to divide participants into placebo analgesia responders and non-responders. Although pain ratings are another, more objective way to measure placebo responding, we decided to stick with these criteria for three reasons: (1) in order to stay consistent with the classification of the here included studies; (2) because those criteria have been successfully employed in our lab in multiple experiments to distinguish responders from non-responders; and (3) because the inclusion of pain ratings as a criterion was hindered by the fact that studies were a mix of between-and within-subject designs. In all studies, participants’ placebo analgesia responses had been assessed using a combination of three main measures, a procedure established as part of the first conducted Study 1 (see Rütgen et al. (2015) for a more detailed description) and used subsequently in all other studies: doubts, beliefs about medication effectiveness, and number of conditioning trials.

First, in every study, doubts that the participants mentioned regarding the deceptive cover story were recorded, specifically about the medication administration and study doctor. In case of serious doubts mentioned independently by the participant throughout the experiment or via follow-up questions after the experiment (e.g., did not believe that they got a real painkiller, believed that the study doctor was fake, etc.), participants were classified as non-responders.

Second, the belief ratings regarding the medication effectiveness obtained before and after the conditioning phase were taken into account. If the total belief was very low (sum of the pre-and post-conditioning rating < 6, on a scale ranging from 1 = “not effective at all” to 7 = “very effective” or its equivalent on a different scale) or if the belief strongly decreased from the first to the second rating (> 3 or its equivalent on a different scale), participants were classified as non-responders.

Third, the number of conditioning trials needed to suggest an analgesic response to the medication was measured. If participants responded with a high rating to the conditioning stimulus delivered with an intermediate intensity, the trial was deemed unsuccessful and repeated after a short waiting period. This procedure was repeated as often as necessary until participants rated the stimuli as intermediate. If four or more of these conditioning trials were needed (in other words, participants still perceived the stimuli as very painful under placebo analgesia), participants were classified as non-responders.

Study 3 used an additional fourth criterion, as this study included a direct comparison of the participants’ hands in a pain task done after the placebo analgesia induction. Pain ratings were compared for the left (control condition) and right (placebo condition) hand. If the average rating of the right hand stimuli was higher than the one for the left hand, participants were classified as non-responders. We thus used the grouping resulting from each study to arrive at our two groups.

### 2.3. Measures

#### 2.3.1. Behavioral data

In each study, participants had completed a battery of questionnaires measuring different dispositional psychological constructs. For this study, we selected all questionnaires measuring empathy and prosocial behavior as well as empathy-related psychiatric conditions, psychopathy, and depression (see Table 1 for an overview of all questionnaires and which were included in which study). Importantly, not all studies administered all questionnaires, leading to different sample sizes in our group analyses (see also Table 2 below). We included a total of 25 subscales out of eight self-report questionnaires (22 out of which were empathy-related), which are detailed below.

The Empathy Components Questionnaire (ECQ; Batchelder et al., 2017) measures cognitive and affective empathy components, but divided into ability and drive components. It consists of five subscales, (1) cognitive ability, the skill, capacity or potential in perspective taking and to adopt another’s point of view; (2) cognitive drive, the motivated interest or tendency in perspective-taking, i.e., to adopt another’s point of view; (3) affective ability, the skill, capacity or potential in recognizing, being sensitive to and sharing others’ emotional experiences; (4) affective drive, the motivated interest or tendency in recognizing, being sensitive to and sharing others’ emotional experiences; and (5) affective reactivity, the emotional response and reaction to other’s emotional experiences. Similar to the QCAE, combining all components yields a cumulative total empathy score. The 27 items are rated on 4-point Likert scales ranging from ‘strongly disagree’ to ‘strongly agree’.

The Empathy for Pain Scale (EPS; Giummarra et al., 2015) assesses empathy specific to seeing another individual in pain. It consists of three subscales: (1) affective distress, feelings of distress, discomfort, fear, avoidance, restlessness, and visceral sensations; (2) empathic concern, feelings of compassion, desire to help, and state empathy; and (3) vicarious pain, feelings of both non-painful and painful vicarious sensations. Across four different scenarios (a person undergoing a surgical procedure, a person who has recently had a surgical procedure, a person being accidentally injured, and a person being physically assaulted), 12 identical items are rated on 5-point Likert scales, ranging from ‘strongly disagree’ to ‘strongly agree’.

The Interpersonal Reactivity Index (IRI; Davis, 1980, 1983) is one of the most frequently used self-report questionnaires to measure empathy. It is a multidimensional measure assessing cognitive and affective empathy across four subscales: (1) Fantasy, the tendency to transpose imaginatively into fictional situations; (2) perspective taking, the tendency to adopt the perspectives of others; (3) empathic concern, the tendency to have feelings of warmth, compassion, and concern for others; and (4) personal distress, the tendency to have feelings of discomfort and anxiety when witnessing others’ negative experiences. The subscales fantasy and perspective taking aim to measure cognitive aspects, whereas the subscales empathic concern and personal distress aim to measure affective aspects of empathy. Each subscale consists of seven items (28 items in total) that are each rated on a 5-point Likert scale ranging from ‘does not describe me well’ to ‘describes me very well’.

The Questionnaire of Cognitive and Affective Empathy (QCAE; Reniers et al., 2011) also provides subscales for cognitive and affective empathy, but draws a more clear distinction between these two components. Cognitive empathy is measured using the subscales (1) perspective taking, the tendency to put oneself in another person’s shoes to see things from his or her perspective and (2) online simulation, the effortful attempt to put oneself in another person’s position by imagining what this other person is feeling. Affective empathy is measured using three subscales: (3) Emotional contagion, the automatic tendency to mirror the feelings of others (4) proximal responsivity, the affective response when witnessing the mood of others in a close social context; and (5) peripheral responsivity, similar to proximal responsivity but in a detached context. Combining all five subcomponents provides a cumulative total empathy score. All 31 items are rated on 4-point Likert scales with response options ranging from ‘strongly disagree’ to ‘strongly agree’.

The Vicarious Pain Questionnaire (VPQ; Grice-Jackson et al., 2017) consists of 16 ten-second videos of people going through painful experiences (such as sports injuries or getting injections; for all video stimuli)^1^ and measures the extent to which participants experience physical sensations of unpleasantness and/or pain when watching these situations. After watching each video, participants are asked to report whether and how intense they experienced pain in their own body on a scale of 1–10, anchored from ‘very mild’ to ‘intense pain’. These two measures are summed up to a total number of pain responses ranging from 0 to 16, and the average pain intensity of those responses. Participants are further prompted to report the localisation of the felt pain and describe their experience using a list of adjectives, including 10 sensory, 10 affective, and 3 cognitive adjectives.^2^ These variables are used to compute two indices regarding localized vs. generalized and sensory vs. affective experiences. Irrespective of whether participants experienced any vicarious pain, the general frequency of such experiences in everyday life is measured on a 10-point Likert scale from ‘hardly ever’ to ‘very regularly’.

The Helping Attitudes Scale (HAS; Nickell, 1998) is a 20-item measure of respondents’ beliefs, feelings, and behaviors associated with helping. Each item is answered on a 5-point Likert scale from ‘strongly disagree’ to ‘strongly agree’ and summed up to one total score.

The Psychopathy subscale of the Short Dark Triad SD3; (Jones and Paulhus, 2013) measures participants’ socially aversive, psychopathic behavior. The nine items sum up to one total score.

Lastly, the revised Beck Depression Inventory (BDI-II; Kühner et al., 2007) assesses acute depressive symptomatology using 21 items corresponding to symptoms of depressions as listed in the Diagnostic and Statistical Manual of Mental Disorders (American Psychiatric Association, 2013). Participants rate the acute severance of the respective symptoms on a 4-point scale, which are then summed up to one total score.

In addition, we used data from the Toronto Alexithymia Scale (TAS; Bagby et al., 1994) and the Autism Quotient (AK-k; Freitag et al., 2007) to check for group differences and possible covariates.

#### 2.3.2. Brain data

For Study 1, structural MRI data were obtained at the Medical University of Vienna, using a 3 Tesla Siemens Tim Trio scanner with a 32-channel head coil. T1-weighted scans of the whole brain were acquired with a magnetization-prepared rapid gradient-echo sequence with the following parameters: TE/TR = 4.21/2,300 ms, 160 sagittal slices, voxel size = 1.0 × 1.0 × 1.1 mm, field of view = 256 mm. In Studies 2 and 3, data were obtained at the University of Vienna’s MRI Center, using a 3 Tesla Siemens MAGNETOM Skyra with a 32-channel head coil. T1-weighted scans of the whole brain were acquired with a magnetization-prepared rapid gradient-echo sequence. Parameters in Study 2 were TE/TR = 2.29/2,300 ms, 176 sagittal slices, voxel size = 0.9 × 0.9 × 0.9 mm, field of view = 240 mm; and in Study 3: TE/TR = 2.43/2,300 ms, 208 sagittal slices, voxel size = 0.8 × 0.8 × 0.8 mm, field of view = 240 mm.

### 2.4. Data analysis

#### 2.4.1. Behavioral data

To analyze the questionnaire data, we performed group comparisons between participants previously classified as placebo responders and non-responders (see 2.6 above). As we had unequal sample sizes (i.e., a higher number of responders compared to non-responders) as well as non-normally distributed data for most questionnaire scales, we used the more robust Welch’s *t*-test for all comparisons (Rasch et al., 2011; Delacre et al., 2017). We also report Cohen’s *d* effect sizes as well as 95% confidence intervals (*CIs*) for all group comparisons. Differences between groups were considered statistically significant for value of *p*s <0.05. For all significant results, we additionally report whether the groups differed in terms of age, gender as well as autistic and depressive traits and the results when including any of those variables into the analysis as a covariate (mean-centered separately for each group). Homogeneity analyses showed that the questionnaire scores were comparable between studies except the localized-generalized dimension of the VPQ (*p* < 0.001) and the Empathic Concern subscale of the IRI (*p* = 0.42). We also exploratorily re-ran our analyses in a linear mixed model with study as a random factor [questionnaire score ~ Responder + (1|Study)]. We further exploratorily conducted outlier identification using the interquartile range criterion (Barbato et al., 2011) and repeated the behavioral analyses without those outliers, which did not change the overall results.

This study was data-driven, in the sense that the initial data were not collected for the purpose of the present study. However, as we tested many questionnaires measuring empathy as a concept, we addressed the issue of type-I error inflation by additionally reporting a correction for multiple comparisons using the Benjamini-Hochberg adjustment (Benjamini and Hochberg, 1995; Benjamini and Yekutieli, 2001), which controls the false discovery rate (FDR). While the commonly used Bonferroni correction is judged as too conservative, especially if hypotheses are non-orthogonal, the Benjamini-Hochberg adjustment is considered as less stringent and thus resulting in more powerful tests (García, 2004; Streiner, 2015; Lee and Lee, 2018; Vander Weele and Mathur, 2019). In this context, the value of *p*s of all empathy-related tests (ECQ, EPS, IRI, QCAE, and VPQ, with a total of 22 subscales/tests) were ordered from the smallest to the largest. Each individual *p* value was then compared to its Benjamini-Hochberg critical value, which was calculated as follows: (rank/total number of tests) x FDR (set at 0.05). The largest value of *p* for which *p* < critical value was classified as significant and all value of *p*s smaller than the largest value were classified as significant as well. For completion, we therefore report and discuss both the uncorrected results of the empathy questionnaires as well as the results after correcting for multiple comparisons. All analyses were conducted in R version 4.1.2.

#### 2.4.2. Brain data

We used FreeSurfer, a well-validated open source software package that uses a surface-based approach to process and analyze structural brain data by creating a three-dimensional model of the cortical surface (versions 6.0.0 and 7.2.0; http://surfer.nmr.mgh.harvard.edu; Rosas et al., 2002; Kuperberg et al., 2003; Fischl, 2012). For preprocessing and analysis, we used the guidelines in the Freesurfer short course provided by Andrew Jahn.^3^ We used Freesurfer’s fully automated standard processing pipeline for anatomical brain data, using the recon-all function. The pipeline consists of multiple stages (for a more detailed description, see Dale et al., 1999; Fischl et al., 1999; Fischl and Dale, 2000).

In brief, T1-weighted images underwent the following steps: skull stripping, intensity normalization, gray-white matter segmentation, and topology correction. Reconstruction of cortical surface models resulted in the gray-white boundary surface as well as the pial surface for each cortical hemisphere. Subcortical regions were segmented, the cortex was parcellated according to the Desikan-Killiany atlas (Desikan et al., 2006), and GMV/CSA/CT as measures of brain morphometry were computed. The resulting maps were smoothed using a 10 mm full-width at half-maximum Gaussian kernel. A total of 174 brains were preprocessed, but reconstruction of one structural image failed due to low image quality resulting from excessive movement during scanning. This participant was excluded, and data analysis was performed on the remaining 173 participants. As the preprocessing pipeline is fully automatic and errors can occur in this process, we conducted an extensive manual quality check procedure and followed the steps described in the tutorial by Andrew Jahn (see link above). We checked both the computed pial and white matter surfaces of the 173 anatomical brain scans by scrolling twice through all brain slices from a coronal view, once focusing on the temporal lobes (as this region is very error-prone) and once focusing on the rest of the brain. If the surface lines were not drawn correctly on three or more consecutive slices, we additionally checked this area in the sagittal and axial view. If the same error was visible in the coronal and one other (sagittal or axial) view, again in at least 3 consecutive slices, we deemed the error meaningful and noted it down. In addition to the two surfaces, we also checked for intensity normalization errors in the white matter. These manual checks were conducted by five individuals (Authors HH and MB as well as three research interns). All quality checkers were trained on five brains that were checked by the main experimenters HH and MB to ensure a homogeneous procedure between individuals, with each brain being checked independently by two of the five people. Non-overlapping errors were discussed until a consensus was reached on whether or not to include those errors and a model-preprocessed brain available in Freesurfer was used for comparison, if needed. After this procedure, major errors were manually corrected using the setting of control points, rerunning parts of recon-all until the error was not visible anymore. Analyses were then performed on the corrected structural brain images.

To examine structural differences between placebo responders and non-responders, we computed a whole-brain analysis for both hemispheres and each outcome measure - GMV, CSA and CT. First, all individual reconstructed cortical surfaces were aligned to an average template using the mri_preproc function in FreeSurfer. Then, intergroup comparisons were performed using vertex-by-vertex general linear models by applying the mri_glmfit function, separate for each hemisphere and measure. Cluster-wise correction for multiple comparisons was performed for all results using pre-computed z Monte Carlo simulations within the function mri_glmfit-sim, which we used to calculate cluster-corrected maps for each contrast. A cluster-wise threshold of *p* < 0.05 was used and the vertex-wise criterion for statistical significance was set at *p* < 0.001 (two-sided test). Results are reported using the contrasts responders vs. non-responders and vice versa.

As two different scanners were used over the whole dataset, we also explored the effect of scanner by including it as a covariate in our whole-brain GLM. We further explored the role of gender as a covariate (for these additional analyses see Supplementary material).

## 3. Results

### 3.1. Behavioral results

**Figure 1:**
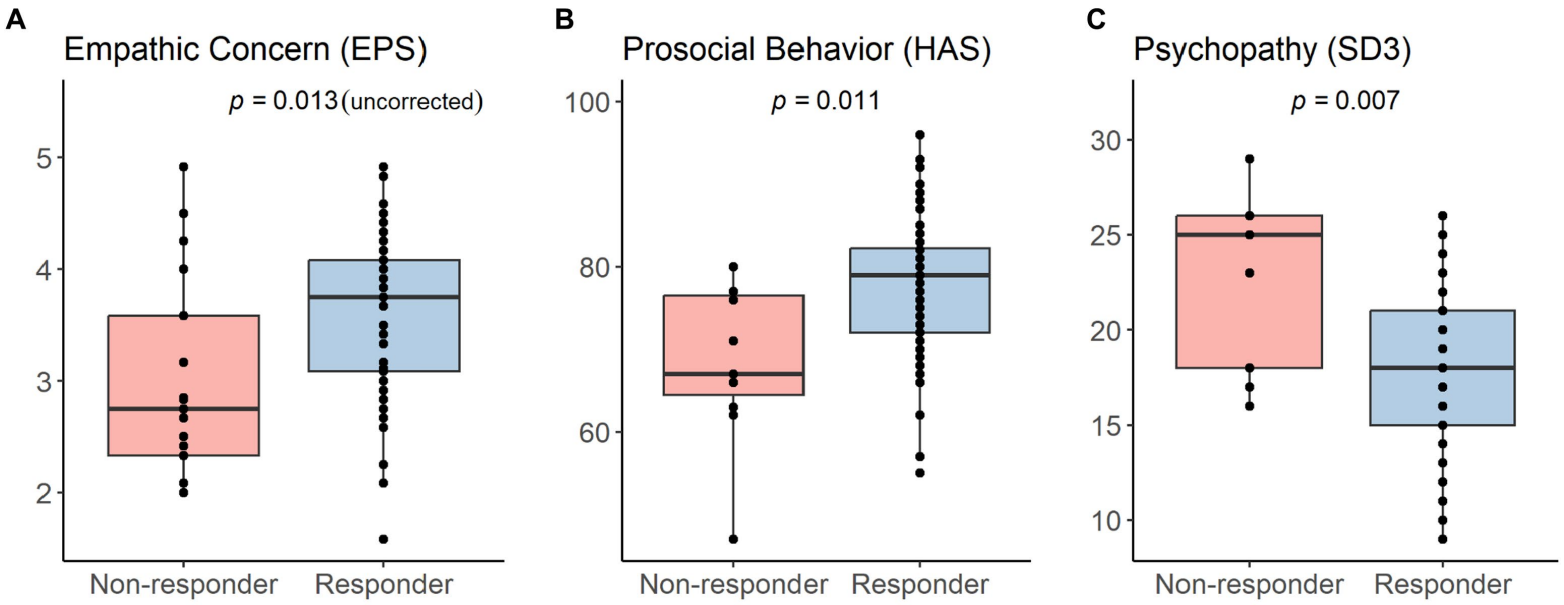
Significant group differences between placebo analgesia responders and non-responders in **(A)** empathic concern (uncorrected, only measured in Study 3), **(B)** prosocial behavior (only measured in Study 4), and **(C)** psychopathy (only measured in Study 4). Correction using the Benjamini-Hochberg adjustment resulted in no significant differences in the empathy subscale **(A)**. Results of all conducted tests including descriptive data and statistics can be found in Table 3.

Table 3 presents the uncorrected results for each questionnaire subscale, which revealed significant group differences in three scales, data of which are displayed in Figure 1 and are reported in the order of the highest to the lowest effect size.

**Table 3:** Group comparisons of personality/questionnaire scores of placebo responders and non-responders.

		R			NR						95% CI			95% CI	
Questionnaire subscale		*n*	*M*	*SD*	*n*	*M*	*SD*	*t* (*df*)	*p*	BH	*LL*	*UL*	Cohen’s *d*	*LL*	*UL*
ECQ^2,3^															
	Affective ability	80	14,96	2,46	31	14,23	2,84	−1.27 (48.42)	0.209	,011	−1,90	0,43	0,28	−0,71	0,14
	Affective drive	80	13,22	1,82	31	12,9	1,3	−1.04 (76.22)	0.302	,020	−0,94	0,30	0,20	−0,60	0,20
	Affective reactivity	80	21,4	2,88	31	21	3,79	−0.53 (44.16)	0.598	,032	−1,92	1,12	0,12	−0,61	0,30
	Cognitive ability	80	18,73	2,41	31	18,06	2,52	−1.26 (52.66)	0.215	,014	−1,72	0,40	0,27	−0,67	0,15
	Cognitive drive	80	16,09	2,19	31	16,03	2,01	−0.13 (59.36)	0.900	,045	−0,93	0,82	0,03	−0,45	0,41
EPS^3^															
	Affective distress	53	2,35	0,72	21	2,27	0,77	−0.45 (34.48)	0.656	,036	−0,48	0,31	0,12	−0,69	0,42
	Empathic concern	53	3,56	0,71	21	2,99	0,89	−2.62 (30.65)	0.013	,002	−1,01	−0,13	0,71	−1,47	−0,17
	Vicarious pain	53	1,95	0,87	21	1,8	0,75	−0.75 (42.39)	0.459	,027	−0,56	0,26	0,19	−0,74	0,27
IRI^1-4^															
	Empathic concern	181	19,8	4,73	56	18,41	5,08	−1.82 (86.52)	0.073	,007	−2,91	0,13	0,28	−0,60	0,00
	Fantasy	181	18,81	5,8	56	18,82	5,23	0.01 (100.50)	0.991	,050	−1,62	1,64	0,00	−0,31	0,32
	Personal distress	181	11,45	4,85	56	10,68	5,01	−1.02 (89.21)	0.311	,023	−2,29	0,74	0,16	−0,45	0,14
	Perspective taking	181	19,12	4,85	56	18,21	4,74	−1.24 (93.46)	0.219	,018	−2,35	0,55	0,19	−0,48	0,12
QCAE^2–4^															
	Emotional contagion	132	10,7	2,4	42	10,43	2,76	−0.58 (61.89)	0.563	,030	−1,22	0,67	0,11	−0,48	0,24
	Online simulation	132	27,23	4,05	42	26,24	4,02	−1.40 (69.60)	0.167	,009	−2,42	0,43	0,25	−0,63	0,10
	Peripheral responsivity	132	10,77	2,77	42	11,21	2,52	0.98 (74.90)	0.330	,025	−0,46	1,36	−0,17	−0,15	0,53
	Proximal responsivity	132	11,61	2,28	42	10,71	2,7	−1.95 (60.83)	0.056	,005	−1,82	0,02	0,36	−0,76	−0,01
	Perspective taking	132	30,77	4,25	42	30,36	5,11	−0.48 (60.11)	,635	,034	−2,16	1,33	0,09	−0,45	0,26
VPQ^3,4^															
	Intensity	86	3,56	1,95	24	3,6	2,05	0.10 (35.44)	,921	,048	−0,90	1,00	−0,02	−0,43	0,45
	Localized-generalized	104	−2,32	5,05	31	−2,1	4,74	0.22 (52.04)	,824	,043	−1,75	2,20	−0,05	−0,36	0,49
	Regularity	82	4,06	1,85	25	3,44	2,26	−1.25 (34.48)	,219	,016	−1,63	0,39	0,30	−0,84	0,16
	Sensory-affective	105	−0,73	10,65	32	−1,31	8,67	−0.31 (62.14)	,756	,039	−4,28	3,12	0,06	−0,46	0,31
	Total pain response	105	5,01	4,51	32	4,72	4,81	−0.30 (48.79)	,763	,041	−2,22	1,64	0,06	−0,52	0.37
HAS^4^															
	Total score	52	78,06	8,98	11	68,64	9,74	−2.95 (13.83)	0.011	---	−16,27	−2,57	1,01	−1,83	−0,41
SD3^4^															
	Psychopathy	52	17,9	4,22	11	22,64	4,52	3.19 (13.92)	,007	---	1,55	7,92	−1,08	0,45	2,01
BDI^2–4^															
	Total score	132	6,45	6,29	42	6,69	5,72	0.23 (75.14)	0.821	---	−1,83	2,31	−0,04	−0,32	0,39

R, placebo analgesia responder; NR, placebo analgesia non-responder; IRI, interpersonal reactivity index; QCAE, questionnaire of cognitive and affective empathy; BDI, beck depression inventory; ECQ, empathy components questionnaire; EPS, empathy for pain scale; VPQ, vicarious pain questionnaire; SD3, short dark triad; HAS, helping attitudes scale; CI, confidence interval; BH, Benjamini-Hochberg critical value. Superscript numbers refer to the studies where a certain questionnaire was used.

First, placebo responders reported to show significantly higher empathic concern specifically related to pain (*M* ± *SD* = 3.56 ± 0.71) than non-responders [*M* ± *SD* = 2.99 ± 0.89; *t* (30.65) = 2.62, *p* = 0.013, *d* = 0.71, 95% *CI* (−1.01, −0.13)] in the EPS. The EPS was only measured in Study 3. Second, placebo responders self-reported significantly higher prosocial tendencies (*M* ± *SD* = 78.06 ± 8.98) than non-responders [*M* ± *SD* = 68.64 ± 9.74; *t* (13.83) = −2.95, *p* = 0.011, *d* = 1.01, 95% *CI* (−16.27, −2.57)] in the HAS. Lastly, we observed that placebo responders reported to show significantly lower psychopathic traits (*M* ± *SD* = 17.90 ± 4.22) compared to non-responders [*M* ± *SD* = 22.64 ± 4.52; *t* (13.92) = 3.19, *p* = 0.007, *d* = −1.08, 95% *CI* (1.55, 7.92)] in the SD3. The HAS and SD3 were only measured in Study 4. In addition, there was a trend for the proximal responsivity subscale of the QCAE, whereby placebo responders reported slightly higher values (*M* ± *SD* = 11.61 ± 2.28) compared to non-responders [*M* ± *SD* = 10.71 ± 2.70; *t* (60.83) = −1.95, *p* = 0.056, *d* = 0.36, 95% *CI* (−1.82, 0.02)]. Importantly, correction for multiple comparisons using the Benjamini-Hochberg adjustment with an FDR of 0.05, revealed no empathy questionnaire value of p smaller than the Benjamini-Hochberg critical value. Thus, no significant group differences in any empathy questionnaire remained after correcting for multiple testing.

With two exceptions, we found no significant group differences regarding age, sex or any autistic/alexithymic/depressive traits in the different questionnaire samples (all *p*’s > 0.079). We did observe a significantly higher amount of females in the IRI responder sample (114 females, 67 males) compared to the non-responder sample (26 females, 30 males) (*p* = 0.041). We also found a significantly higher mean age in responders (*M* ± *SD* = 23.94 ± 2.75) compared to non-responders (*M* ± *SD* = 22.38 ± 2.56) in the EPS sample (*p* = 0.026). However, this mean difference of 1.56 years was relatively small and likely negligible. Including gender, age, autistic traits, depressive traits, or alexithymic traits separately as covariates did not change the presented results. The exploratory linear mixed model with study as a random factor revealed the same results as above, but additional group differences in two empathy questionnaires, the Empathic Concern scale of the IRI (*p* = 0.045) and the Proximal Responsivity scale of the QCAE (*p* = 0.028). In both cases, non-responders had lower scores than responders. However, these two results did not survive the BH correction for multiple comparisons.

### 3.2. Brain results

**Figure 2:**
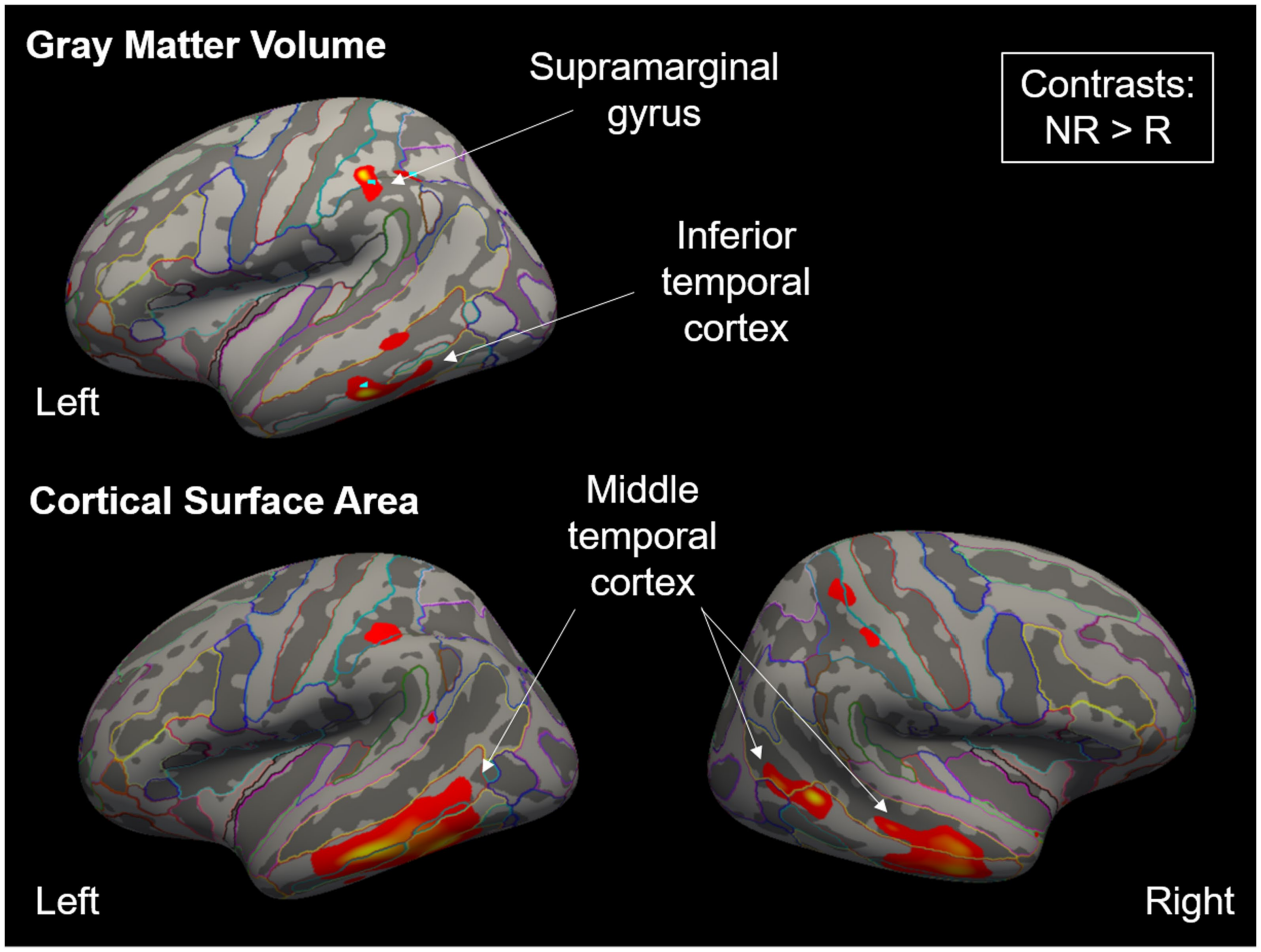
Significant clusters for gray matter volume and cortical surface area when comparing placebo analgesia non-responders (NR) to responders (R), displayed as a heatmap. Clusters were significant for NR > R, while the opposite contrasts did not show significant differences. Colorful outlines show parcellation using the Destrieux atlas (Destrieux et al., 2010). When re-running the whole-brain GLMs using total intracranial volume as a covariate, no result remained significant at the chosen cluster-wise correction level.

Next, we investigated structural brain differences on the whole-brain level between the two groups in our aggregated sample of 173 individuals (see Table 4; Figure 2). The responders and non-responders of the brain data sample did not differ significantly regarding age, gender or any autistic/alexithymic/depressive traits (*p*’s > 0.100), but they did differ significantly in their total intracranial volume [TIV; *t* (70.71) = 2.57, *p* = 0.012], their white matter volume [*t* (77.59) = 2.33, *p* = 0.022], and their cerebrospinal fluid volume [*t* (66.85) = 2.19, *p* = 0.032]. As these differences make the inclusion of them as covariates difficult (Miller and Chapman, 2001), but especially correction for TIV is routinely done in studies assessing structural brain measures such as CSA and GMV that scale with TIV, we therefore report whole-brain results with and without correction for TIV.

**Table 4:** Structural brain differences for the contrast placebo analgesia non-responders > responders without covariates.

Measure and brain region	h	VtxMax	size	*x*	*y*	*z*	*p*	CI (*p*)
GMV								
Inferior temporal	L	120,836	338.40	−54.0	−33.6	−16.9	0.003	[0.002, 0.004]
Supramarginal	L	123,297	249.71	−44.3	−32.2	34.6	0.017	[0.013, 0.017]
CSA								
Middle temporal	R	22,997	1259.90	61.3	−14.3	−21.9	< 0.001	[< 0.0001, 0.0004]
Middle temporal	R	91,021	452.16	57.9	−57.4	5.0	0.010	[0.008, 0.012]
Middle temporal	L	49,559	2389.60	−59.7	−28.6	−16.8	< 0.001	[< 0.0001, 0.0004]
CT								
No significant clusters								

Significant clusters separate for gray matter volume (GMV), cortical surface area (CSA) and cortical thickness (CT), including hemisphere h, vertex number at maximum (VtxMax), cluster surface area in mm^2^ (size), MNI coordinates x, y, z, cluster-wise value of *p* (threshold of *p* < 0.05, vertex-wise criterion for statistical significance at *p* < 0.001, two-sided) and the 90% confidence intervals (CI) of that *p*-value.

In the contrasts non-responder vs. responder, we found increased GMV in left inferior temporal and supramarginal cortices (two clusters) and increased CSA in bilateral middle temporal cortex (three clusters), while there were no differences between the groups regarding CT. We did not find any significant differences in any of the opposite contrasts (responder > non-responder) in any of the three measures. Importantly, when re-running the whole-brain GLMs using TIV as a covariate, no result remained significant at the chosen cluster-wise correction level. Exploratory analyses including scanner or gender as a covariate are reported in the Supplement.

## 4. Discussion

The aim of the present study was to investigate potential differences in psychological traits and brain structure between placebo analgesia responders and non-responders, using a mixed confirmatory and exploratory approach and focusing on social psychological factors. Prior research on the link between psychological characteristics and placebo responding has found few reliable results. In order to provide more clarity and robust results, the current study intended to overcome the limitations of earlier studies by using a larger sample size and stable context factors. Our findings revealed significant differences in empathy-related trait constructs, with placebo responders reporting increased prosocial tendencies, and lower psychopathic traits compared to non-responders. We also observed higher pain-related empathic concern in responders vs. non-responders, although this did not withstand correction for multiple comparisons. Additionally, the neuroimaging data demonstrated smaller gray matter volume and smaller cortical surface area in placebo analgesia responders in supramarginal gyrus, as well as inferior and middle temporal gyrus.

Regarding the behavioral data, placebo responders self-report indicated they display more prosocial and less psychopathic traits, compared to non-responders. Furthermore, responders reported higher empathic concern for the pain of others (uncorrected), while we did not find such group differences in our other measures of general empathy. Interestingly, the pain-specific empathic responses were related to feeling compassion and the need to help others. Even though, this result was non-significant after correcting for multiple comparisons, our results are in line with previous literature on the relationship between these concepts, showing a positive relationship between compassion and prosociality (Karnaze et al., 2022), as well as a negative relationship between social emotions or behavior and psychopathy (Van Dongen, 2020). However, it is important to note that these three results were the only significant ones out of a total of 25 conducted tests. When correcting for 22 empathy-related tests, the empathic concern scale did not reach statistical significance anymore. It is also interesting to note that the other two findings were adjacent but different constructs, prosocial behavior and psychopathic traits. These data thus give hints which questionnaires might be worth further investigation in future studies with larger samples.

The placebo response is a highly complex phenomenon, which is based on a multitude of factors such as person, situation, and/or context. Past research demonstrated that depending on the used paradigm/context, results could vary greatly, and in some cases, even lead to a reversed association between personality traits and the placebo response. For instance, a negative relationship between empathy and the benefit of the placebo treatment was found in a stress paradigm (Darragh et al., 2014), contrasting the frequently reported positive relationship in pain paradigms (Colloca and Benedetti, 2009; Hunter et al., 2014). Similar observations have also been reported for several other personality traits, such as extraversion and optimism (Darragh et al., 2014). Overall, the link between personality and placebo responding is not straightforward but instead might be modulated by environmental factors such as context and/or situation. Thus, it seems highly probable that, depending on the context, different personality traits become more or less relevant for the placebo response (Whalley et al., 2008; Darragh et al., 2015). In our case, pain-related empathic traits appear to be characteristics in which placebo responders differ from non-responders specifically inside of highly similar pain paradigms. However, this study is the first that included a pain-specific empathy measure so far; therefore, this preliminary assumption needs further investigation. Nevertheless, in order to harness the full potential of the “placebo personality,” e.g., to utilize personality traits in order to predict future placebo responding, it might be beneficial to match personality traits and the context of placebo administration.

By systematically including context factors, future studies could further elucidate the question under which circumstances, which personality traits are important for the placebo response. Moreover, it could help identify traits which are stable across different contexts and traits which are changing depending on the context. One such context factor to consider is the relationship quality between healthcare practitioner and patient. It has repeatedly been shown to have a significant influence on the placebo response, whereby factors such as empathy, warmth, communication of positive expectations, and duration of interaction enhance the magnitude of the placebo response (Kaptchuk et al., 2008; Kelley et al., 2009). As none of our included studies measured this variable, this hypothesis remains speculative and future studies could for example measure both patients’ and clinicians’ empathic abilities and investigate their additive vs. distinct association with social abilities.

In relation to this, our findings also have implications for the translation of artificial lab experiments to real-world settings such as the doctor’s office, where individuals may have different motivations (pleasing the experimenter vs. getting help) or need for relief (e.g., no prior complaints vs. symptom reduction). Future studies in this field should thus shift focus towards answering the question, whether predictors of placebo responsiveness in the lab can be readily transferred to the real world, and if not, how they can be made more useful. The answer to this question may have profound consequences for future research.

In accordance with Coll et al. (2017) we consider empathy as the product of two processes, namely identification of another’s emotional state (emotion identification) and sharing the other’s emotional state (affect sharing). Even though the amount of empathy an individual has towards another is highly context dependent, there is interindividual variability in how well these two processes are performed. For example, one might demonstrate a lower empathic response due to inadequate emotion identification or due to a limited ability to share the affective state of another. Consequently, in this conceptual frame, some individuals can have higher trait empathy than others by performing better at identifying and sharing another’s affective state. Now, our present results imply a positive effect of higher empathy levels, and higher prosocial tendencies in general, on placebo responsiveness. So why could higher prosocial traits lead to enhanced placebo responses? First of all, it needs to be considered that empathy plays an essential role in building relationships. Additionally, the quality of the physician-patient relationship is of particular importance for the treatment outcome. In this respect, a study has demonstrated that the physician’s ability to empathize with the patient in combination with the patients’ dispositional optimism led to significantly improved health outcomes in chronic pain patients (Cánovas et al., 2018). Furthermore, higher interpersonal trust in the dyadic physician patient relationship has demonstrated similar positive effects (Birkhäuer et al., 2017; Wu et al., 2022). Since earlier lines of research have linked higher dispositional optimism to higher placebo responsiveness (Morton et al., 2009; Geers et al., 2010) we suggest that these positive effects on the placebo response could be explained by the overall emotional valence of the dyadic interaction: traits which have beneficial effects on interpersonal interactions, such as empathy, prosocial traits, optimism, and the absence of obstructive factors (e.g., psychopathy) favor an emotional environment, which facilitates the elicitation of high placebo responses. Moreover, the better the match between personality traits and contextual factors (e.g., pain study paradigm), the higher the chance for a placebo response. In line with this argument, using participants’ empathy for other people’s pain might be an interesting avenue for predicting their individual hypoalgesic response to a placebo. However, it is important to be cautious with this interpretation as the empathy results were non-significant after correction for multiple comparisons and this should be regarded as preliminary, and in need of replication and extension.

Regarding the brain data, we found smaller gray matter volume and smaller cortical surface area in placebo analgesia responders compared to non-responders in the supramarginal gyrus, as well as the inferior and middle temporal gyrus. Given that placebo responses are the result of complex central nervous system mechanisms, including expectancies and different types of learning processes (Benedetti, 2013), we assumed that measures of brain structure would be able to distinguish placebo responders from non-responders. However, the found regions do not match with the few previous studies, which highlight a positive correlation of gray matter density in brain areas such as the dorsolateral prefrontal cortex, insula, and nucleus accumbens and placebo analgesic effects (Schweinhardt et al., 2009).

Earlier studies suggest that larger cortical surface area is related to higher overall neurocognitive performance whereas smaller cortical surface area is associated with problems in executive functioning (Gutiérrez-Galve et al., 2010; Hartberg et al., 2011; Tamnes et al., 2015; Tadayon et al., 2020). This can be explained by the circumstance that a greater cortical surface area corresponds to a more convoluted sulcal shape with a greater amount of corticocortical connections, which drives local processing. This might increase the performance of brain function and improve cortical computation (Im et al., 2008). Our placebo non-responders demonstrated higher cortical surface area in several regions compared to placebo responders, which somewhat contradicts our prior assumptions. However, the observed increase in cortical surface area in non-responders could represent an underlying opposing mechanism, which hampers the formation of placebo responses. Thus, higher cortical surface area in these regions could be associated with lower expectancies, empathy (as demonstrated in our results), and overall traits which are detrimental for the placebo response, leading to lower placebo responses. What has to be noted, though, is the absence of typically found regions related to the neural basis of empathy. Previous work highlights negative associations of general empathic traits with gray matter volume in precuneus inferior frontal gyrus, anterior cingulate cortex (Banissy et al., 2012) as well as anterior insula (Li et al., 2020). We, on the other hand, found structural brain differences between responders and non-responders in middle and inferior temporal areas. Thus, the relationship of placebo responsiveness and empathy on the neural level will have to be investigated further in the future.

It is possible that limitations might have influenced our results and therefore, a number of shortcomings should be acknowledged. One caveat of the present study is that our study was exploratory, and that some of the analyses do not withstand correction for multiple comparisons, and the brain results do not hold when correcting for total intracranial volume (TIV). This means that our brain differences could also be the result of inherent brain size differences between the groups. As our two groups differed significantly in their TIV, inclusion of this variable as a covariate becomes difficult. Interestingly, exploratory analyses showed that brain results differed when including gender as a covariate. Moreover, including scanner as a covariate changed the resulting clusters, while identifying similar regions largely located in the temporal cortex. Both of these analyses highlight the need to include them as a covariate in future studies (for gender effects in placebo research see, e.g., Enck and Klosterhalfen, 2019). Especially because of this, replication and theoretical extension of our findings is needed. Even when using a pooling approach spanning multiple studies and thus increasing the sample size, like the one used here, power may still be too low to detect these subtle differences using stricter corrections. Future work in this field should therefore focus on using a much larger sample of participants, for example data repositories like the Human Connectome Project^4^ and NeuroVault^5^, or collaborate in multi-lab studies (see, e.g., the ManyLabs projects) to investigate this and similar research questions.

Furthermore, we used a convenience sample, pooling data from four of our own lab’s placebo studies. An effort towards validation and generalization of the present findings could be achieved by including placebo analgesia studies from multiple labs and even countries. The included studies all come from the field of placebo analgesia research and utilized a similar study design, yet for the present research question, this design could be deficient itself, as it assessed the placebo response only once. For the interpretation of our results, the placebo response was seen as a stable trait of an individual. In other words, subjects who responded to the placebo, are expected to demonstrate this response in the future as well. Several papers have addressed this issue and suggest temporal stability, given that contextual factors remain stable (Whalley et al., 2008; Ashar et al., 2017). Since the temporal stability of the placebo response is an important prerequisite to harness the response for other purposes (e.g., the adjustment of a medical treatment based on an individual’s tendency to respond to a placebo), future studies should aim to address this matter in their study designs. Relatedly, we chose to employ *a-priori* non-responder classification criteria of the included studies as opposed to pain ratings. Future work should investigate direct comparisons of certain criteria and which ones, alone or in combination, best predict placebo (non)responding. Lastly, using previously collected convenience samples meant that not all studies obtained the same trait measures, which is why sample sizes differ in our questionnaire analyses, and especially results from smaller samples should be interpreted with caution until independently replicated.

As evident from the present findings, it remains a challenge to narrow down on a few selected predictive criteria that are able to predict the placebo response. Nevertheless, our study adds valuable information on additional possible social characteristics contributing to the existing evidence. We also propose that placebo responder research needs to move away from the intention to identify single traits which are exclusively related to the magnitude of the placebo response and towards a larger context. Future studies should pursue an interactional approach, by including context-specific factors as well as a large range of psychological and biological characteristics. In this way, context-specific placebo responder patterns can be identified.

Overall, the present study does not provide clear-cut generalizable evidence but draws a heterogenous picture in terms of placebo responder characteristics. Our results broaden the understanding of placebo responder characteristics inside of pain contexts and additionally, we highlight new directions towards social emotions and behavior for exploring differences between responders and non-responders. Importantly, we also provide important hints where future studies could look for biomarkers regarding placebo responsiveness in the brain. While the question “Who responds to placebos?” is still only partly answered, the present study brings us one step closer to answering this crucial question, and paves the way for a better identification of placebo responders in clinical contexts.

## Data availability statement

The datasets presented in this study can be found in online repositories. The names of the repository/repositories and accession number(s) can be found at: https://osf.io/nwgdj/?view_only=d3d8e54e20f4453fba0ea10b878bb0f3.

## Ethics statement

The studies involving humans were approved by the ethics committee of the Medical University of Vienna (Study 1, Study 2, and Study 3), and the University of Vienna’s ethics committee (Study 4). All studies were performed in accordance with the Declaration of Helsinki (2013). The studies were conducted in accordance with the local legislation and institutional requirements. The participants provided their written informed consent to participate in this study.

## Author contributions

HH: Conceptualization, Data curation, Formal analysis, Funding acquisition, Investigation, Methodology, Project administration, Software, Supervision, Visualization, Writing – original draft, Writing – review & editing. MB: Data curation, Formal analysis, Investigation, Methodology, Software, Visualization, Writing – original draft, Writing – review & editing. FR: Conceptualization, Investigation, Methodology, Project administration, Supervision, Writing – review & editing. CL: Conceptualization, Funding acquisition, Resources, Supervision, Writing – review & editing.

## Funding

The author(s) declare financial support was received for the research, authorship, and/or publication of this article. The present study was financially supported by the uni:docs scholarship of the University of Vienna, the Marietta-Blau scholarship of the Agency for Education and Internationalisation Austria (OeaD) (both to HH), and the Austrian Science Fund (FWF W1262-B29 and P32686 to CL). None of the funders had any role in study design, data collection and analysis, interpretation, writing or decision to publish.

## Conflict of interest

The authors declare that the research was conducted in the absence of any commercial or financial relationships that could be construed as a potential conflict of interest.

## Publisher’s note

All claims expressed in this article are solely those of the authors and do not necessarily represent those of their affiliated organizations, or those of the publisher, the editors and the reviewers. Any product that may be evaluated in this article, or claim that may be made by its manufacturer, is not guaranteed or endorsed by the publisher.

## Footnotes

^1^See https://www.youtube.com/channel/UCT8goTgWGRsu14NjVaPCSGw/videos.

^2^See https://youtu.be/iYkVmLfAt6w for more details on the analysis and score calculation.

^3^Andy’s Brain Book, https://andysbrainbook.readthedocs.io/en/latest/FreeSurfer/FreeSurfer_Introduction.html.

^4^ https://www.humanconnectome.org/

^5^ https://neurovault.org/
