# Supplementary material for: To respond or not to respond: exploring empathy-related psychological and structural brain differences between placebo analgesia responders and non-responders
Source: Front Psychol. 2023 Oct 2;14:1257522. doi: 10.3389/fpsyg.2023.1257522 (PMC10577216; doi:10.3389/fpsyg.2023.1257522)
Supplement: Supplementary file 1 [file Table_1.DOCX]

Supplement to

**To respond or not to respond: Exploring empathy-related psychological and structural brain differences between placebo analgesia responders and non-responders**

**Helena Hartmann^1,2*^, Magdalena Banwinkler^1,3*^, Federica Riva^1*^, & Claus Lamm^1^**

^1^ Social, Cognitive and Affective Neuroscience Unit, Department of Cognition, Emotion, and Methods in Psychology, Faculty of Psychology, University of Vienna, Vienna, Austria

^2^ Clinical Neurosciences, Department of Neurology, University Hospital Essen, Essen, Germany

^3^ Faculty of Medicine and University Hospital Cologne, Department of Nuclear Medicine, University of Cologne, Cologne, Germany

^*^ These authors contributed equally to this work and share first authorship.

**Effects of scanner**

When including scanner as a covariate, the identified clusters changed, but were still largely located in temporal areas (e.g., inferior and middle temporal cortex for gray matter volume (GMV), and inferior, middle and superior temporal cortex as well as fusiform gyrus for cortical surface area (CSA; see Table S1).

| Table S1  *Structural brain differences for the contrast placebo analgesia non-responders > responders, after including scanner as a covariate.* | | | | | | | | |
| --- | --- | --- | --- | --- | --- | --- | --- | --- |
| Measure and brain region | h | VtxMax | size | *x* | *y* | *z* | *p* | CI (*p*) |
| **GMV** |  |  |  |  |  |  |  |  |
| Inferior temporal | L | 25455 | 210.51 | -55.1 | -32.5 | -16.6 | .022 | [.019; .025] |
| Middle temporal | R | 107870 | 324.36 | 47.2 | -28.4 | -9.9 | .001 | [< .001; .002] |
| Middle temporal | R | 115131 | 208.81 | 56.5 | -13.1 | -24.9 | .027 | [.024; .030] |
| **CSA** |  |  |  |  |  |  |  |  |
| Inferior temporal | L | 114325 | 2103.58 | -45.8 | -49.6 | -12.6 | < 001 | [< .0001; .0004] |
| Superior temporal | L | 147042 | 423.70 | -59.5 | -50.3 | 15.8 | .014 | [.012; .017] |
| Superior temporal | L | 89973 | 370.37 | -53.0 | 6.5 | -13.0 | .026 | [.026; .029] |
| Middle temporal | R | 46526 | 1446.71 | 59.5 | -15.2 | -17.5 | < .001 | [< .0001; .0004] |
| Fusiform | R | 65080 | 444.12 | 38.3 | -67.9 | -14.3 | .011 | [.009; .013] |
| **CT** |  | | | | | | | |
| No significant clusters | | | | | | | | |
| *Note.* Significant clusters separate for gray matter volume (GMV), cortical surface area (CSA) and cortical thickness (CT), including hemisphere h, vertex number at maximum (VtxMax), cluster surface area in mm^2^ (size), MNI coordinates x, y, z, cluster-wise *p*-value (threshold of *p* < .05, vertex-wise criterion for statistical significance at *p* < .001, two-sided) and the 90% confidence intervals (CI) of that *p*-value. | | | | | | | | |

**Effects of gender**

When including gender as a covariate, only one cluster in the lateral occipital cortex both for GMV and CSA remained (R > NR; see Table S2). Importantly, this region had larger volume and surface area, but for responders in comparison to non-responders.

| Table S2  *Structural brain differences for the contrast placebo analgesia responders > non-responders, after including gender as a covariate.* | | | | | | | | |
| --- | --- | --- | --- | --- | --- | --- | --- | --- |
| Measure and brain region | h | VtxMax | size | *x* | *y* | *z* | *p* | CI (*p*) |
| **GMV** |  |  |  |  |  |  |  |  |
| Lateral occipital | L | 126649 | 353.08 | -27.9 | -87.1 | -0.6 | < .001 | [.0004; .001] |
| **CSA** |  |  |  |  |  |  |  |  |
| Lateral occipital | L | 52330 | 734.01 | -27.4 | -90.5 | -1.0 | < .001 | [< .0001; .0008] |
| **CT** |  | | | | | | | |
| No significant clusters | | | | | | | | |
| *Note.* Significant clusters separate for gray matter volume (GMV), cortical surface area (CSA) and cortical thickness (CT), including hemisphere h, vertex number at maximum (VtxMax), cluster surface area in mm^2^ (size), MNI coordinates x, y, z, cluster-wise *p*-value (threshold of *p* < .05, vertex-wise criterion for statistical significance at *p* < .001, two-sided) and the 90% confidence intervals (CI) of that *p*-value. | | | | | | | | |
